# Supplementary material for: Click Chemistry with Cell-Permeable Fluorophores Expands the Choice of Bioorthogonal Markers for Two-Color Live-Cell STED Nanoscopy
Source: Cells. 2024 Apr 15;13(8):683. doi: 10.3390/cells13080683 (PMC11049381; doi:10.3390/cells13080683)
Supplement: Supplementary file 1 [file cells-13-00683-s001.zip › cells-2879994 Supporting Information.pdf]

Supporting Information

Table S1. Primers used for plasmid construction.

Primer name	Sequence (5'→3')
NES-hMbPylRS NheI fwd	TGTTAGGCTAGCGCCACCATGGCCTGCCCCGTGCCCCTG-<br>CAGCTGCCCCCCCTGGAGCGCCTGACCCTGGACGACAAG-<br>AAACCCCTGGACGT
hMbPylRS NotI rev	TTGATAGCGGCCGCTCACAGGTTGGTGGAGAT
ACTB NheI fwd	TTGATCGCTAGCATGGATGATGATATCGCCGC
ACTB BamHI rev	GTTATCGGATCCTCAGAAGCATTTGCGGTGGA
ACTB K118TAG	[Phos]-CCAAGGCCAACCGCGAGTAGATGACCCAGATCATG
ACTB BamHI fwd	CGATATCGCGGATCCGATGATGATATCGCC
ACTB NotI rev	ATAGTTTAGCGGCCGCCTAGAAGCATTTGCG
EGFP NheI fwd	TGAATAGCTAGCATGGTGAGCAAGGGCGAG
EGFP HindIII rev	GATCCCAAGCTTCTTGTACAGCTCGTC
mNeptune2 NheI fwd	TGAATGGCTAGCATGGTGTCTAAGGGCGAA
mGarnet NheI fwd	TGTAATGCTAGCATGAACAGCCTGATCAAA
mGarnet HindIII rev	GTTATAAAGCTTCCCTCCGCCCAGGCCGGC

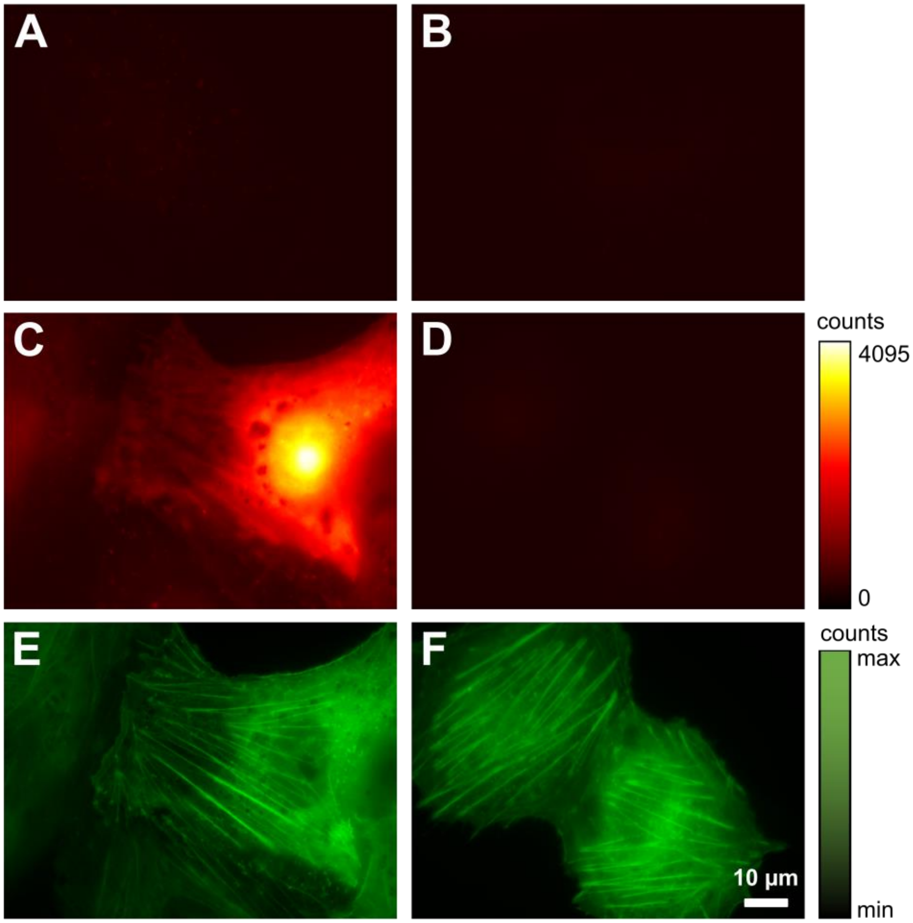

Figure S1. Comparison of background signal in living CV-1 cells with and without expression of tRNA/tRNA synthetase and incubation with TCO*A. **(A,B)** Untransfected cells were incubated with (A) or without TCO*A (B) before labeling with SiR-tetrazine. **(C,D)** Cells were transfected with plasmids encoding the tRNA/tRNA synthetase and EGFP-actin and incubated with (C) or without TCO*A (D) before labeling with SiR-tetrazine. The same colormap was used for all images in A–D. **(E,F)** Fluorescence of EGFP-actin of the same cells as shown in (C,D) as a control for cellular transfection.

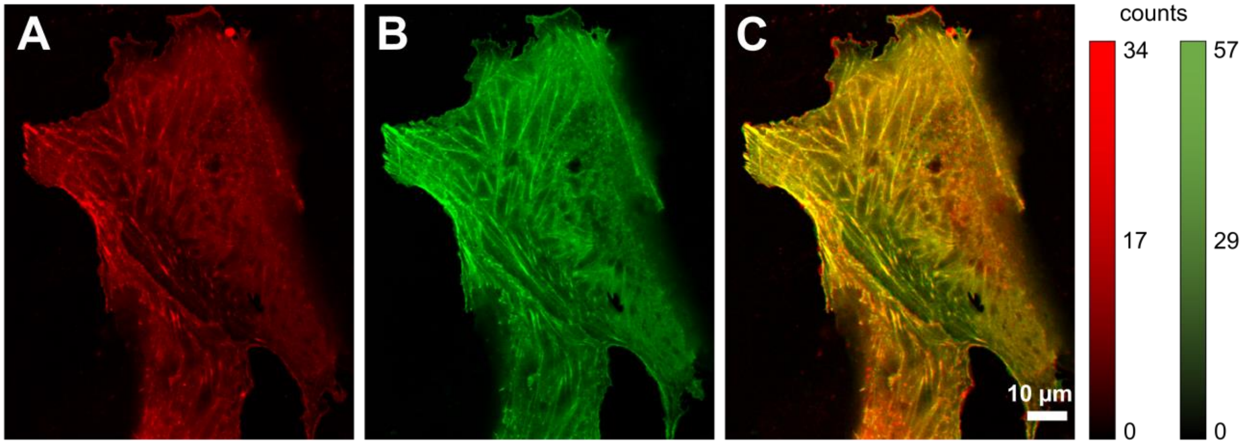

Figure S2. Actin structures of a living CV-1 cell. Cells were cotransfected with plasmids encoding EGFP-actin and actin^{K118TAG} and incubated with TCO*A. The cells were labeled with LIVE 610 click and imaged by confocal microscopy. **(A)** Fluorescence of LIVE 610 excited at 640 nm. **(B)** Fluorescence of EGFP excited at 488 nm. **(C)** Superposition of (A) and (B).

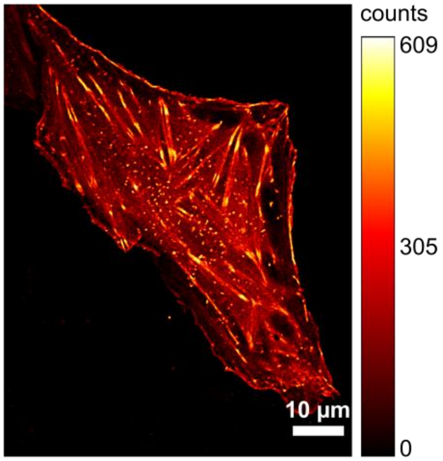

Figure S3. Fluorescence imaging of actin filaments click-labeled with LIVE 510. Confocal image of a living CV-1 cell expressing actin^{K118TAG} which was labeled with LIVE 510 click. The fluorophore was excited with a 488 nm laser.

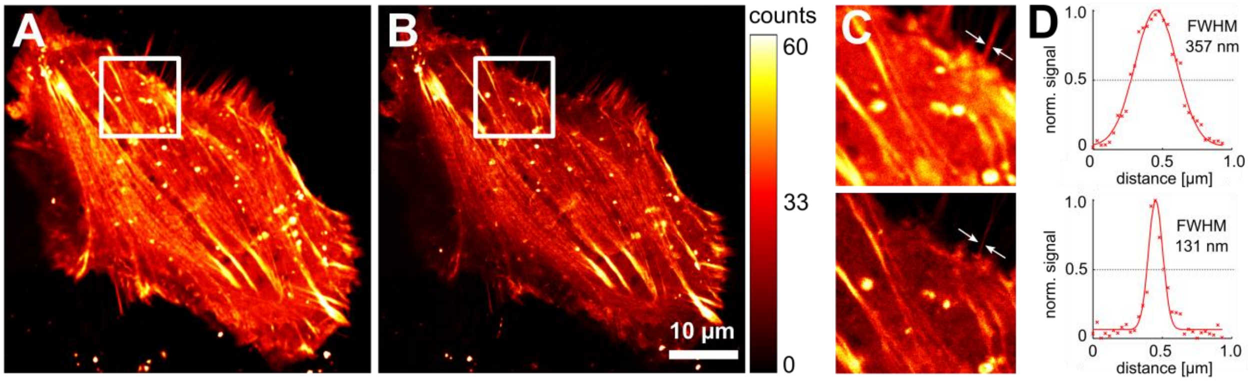

Figure S4. Fluorescence imaging of actin filaments click-labeled with LIVE 460L. **(A)** Confocal and **(B)** STED image of a living CV-1 cell expressing actin^{K118TAG} which was labeled with LIVE 460L click. **(C)** The upper panel shows a close-up of the confocal image (A), while the lower panel shows the close-up of the STED image (B). **(D)** Line profiles of the filament marked with an arrow in the confocal (upper panel) and STED image (lower panel) of (C).The fluorophore was excited with a 488 nm laser. For STED imaging, a 775 nm depletion laser was used.

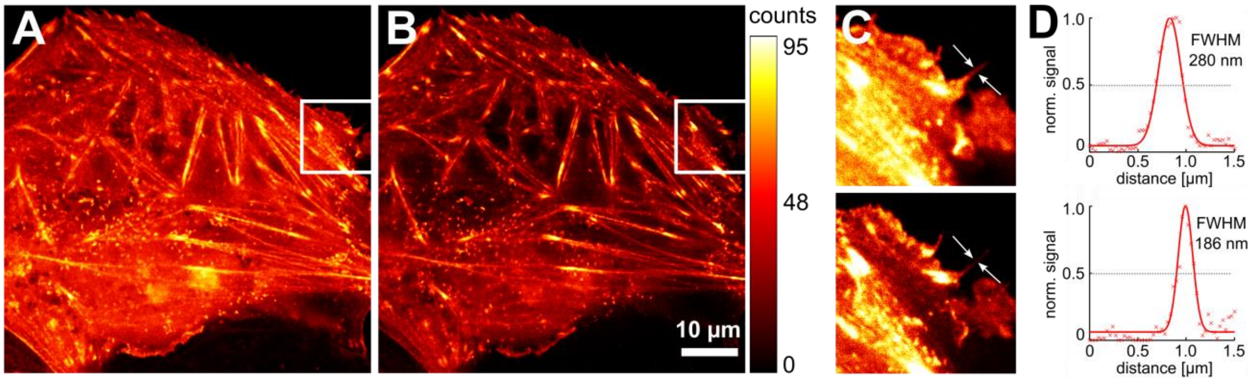

Figure S5. Fluorescence imaging of actin filaments click-labeled with LIVE 550. **(A)** Confocal and **(B)** STED image of a living CV-1 cell expressing actin^{K118TAG} which was labeled with LIVE 550 click. **(C)** The upper panel shows a close-up of the confocal image (A), while the lower panel shows the close-up of the STED image (B). **(D)** Line profiles of the filament marked with an arrow in the confocal (upper panel) and STED image (lower panel) of (C).The fluorophore was excited with a 561 nm laser. For STED imaging, a 775 nm depletion laser was used.

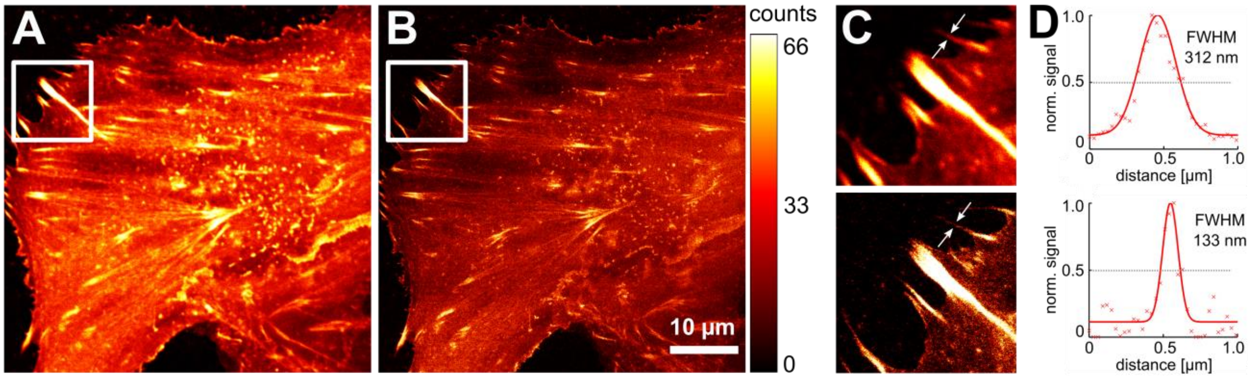

Figure S6. Fluorescence imaging of actin filaments click-labeled with LIVE 610. **(A)** Confocal and **(B)** STED image of a living CV-1 cell expressing actin^{K118TAG} which was labeled with LIVE 610 click. **(C)** The upper panel shows a close-up of the confocal image (A), while the lower panel shows the close-up of the STED image (B). **(D)** Line profiles of the filament marked with an arrow in the confocal (upper panel) and STED image (lower panel) of (C).The fluorophore was excited with a 640 nm laser. For STED imaging, a 775 nm depletion laser was used.

Video S1. Two-color long-term STED imaging of actin and mitochondria. Living CV-1 cells expressing actin^{K118TAG} and OMP25-SNAP were incubated with TCO*A and labeled with LIVE 550 click and LIVE 610 SNAP. The dyes were excited with a 561 nm and a 640 nm laser, respectively, and depleted with a 775 nm STED laser. Images were recorded every 30 s for a period of 30 min.
